# Two case reports: Early detection of amniotic band syndrome by adhesion between hand and umbilical cord at 11 to 14 weeks’ gestation

**DOI:** 10.1097/MD.0000000000018302

**Published:** 2019-12-16

**Authors:** Zihan Niu, Hua Meng, Xiaoyan Zhang, Yunshu Ouyang, Yixiu Zhang, Xining Wu

**Affiliations:** a Department of Ultrasound, Peking Union Medical College Hospital, Chinese Academy of Medical Sciences and Peking Union Medical College; b Beijing Dongcheng First Maternal & Child Health Hospital, Beijing, China.

**Keywords:** adhesion between hand and umbilical cord, amniotic band syndrome, umbilical cord

## Abstract

**Rationale:**

The significant ultrasonic characteristics of amniotic band syndrome (ABS) are the malformations of fetal affected parts and the band—like echoes in amniotic cavity. This article first suggests that the fetal hand adhered to umbilical cord with restricted movement provides some values in the diagnosis of ABS in early gestational weeks especially when the fetal malformation is not obvious and amniotic band is thin and fine.

**Patient concerns:**

Two pregnant women had no discomfort and underwent routine ultrasound examination at 11 to 14 gestational weeks.

**Diagnosis:**

Only the fetal hand adhered to umbilical cord with restricted movement was detected during the first ultrasound examination at 11∼14 gestational weeks, and the floating band-like echos were detected in the amniotic cavity with follow-up examinations 2 to 3 weeks later. Both of the 2 fetus were diagnosed as ABS by ultrasound

**Interventions:**

The two pregnant women underwent the prenatal counseling and were recommended closely follow-up and further examination.

**Outcomes:**

Two fetuses died in utero between 17 and 19 weeks. After induction of labor, it was found that the hands and umbilical cord of the fetuses were wrapped by amniotic bands, which was proved pathologically as ABS.

**Lessons:**

The adhesion of the fetal hand and umbilical cord is an important ultrasonic sign suggesting ABS with poor prognosis in early pregnancy. We hope that this study can provide some guidance for the early diagnosis of ABS during 11 to 14 week's ultrasound examination.

## 1 Introduction

Amniotic band syndrome (ABS) with complex etiology is a rare and serious congenital anomaly of the fetus and appendages, in which the fetal body parts are tangled or wrapped by ruptured amniotic bands, resulting in fetal structural abnormalities and dysfunctions. Prenatal diagnosis of ABS mainly depends on fetal ultrasound (US) examination. The abnormalities of fetal affected parts and the band—like echoes in amniotic cavity—are suggestive for ABS. Usually, the amniotic band is thin and the fetal malformation is not obvious in early gestational weeks, which increase the difficulty of ABS diagnosis. In our 2 cases of ABS, only the fetal hand adhered to umbilical cord with restricted movement was detected during the first transabdominal-ultrasound examination at 11∼14 gestational weeks, and the floating bands in the amniotic cavity were found 2 to 3 weeks later. Both fetuses died in the second trimester of pregnancy. As far as we know, this is the first report that ABS in early pregnancy can be characterized by adhesion between fetal hand and umbilical cord with limited movement. Therefore, it is of great significance to observe the relationship between the umbilical cord and the hand at 11∼14 weeks for the diagnosis and prognosis of ABS. The US characteristics and follow-up results were analyzed and summarized.

## 2 Case study

### 2.1 Case 1

**Figure 1:**
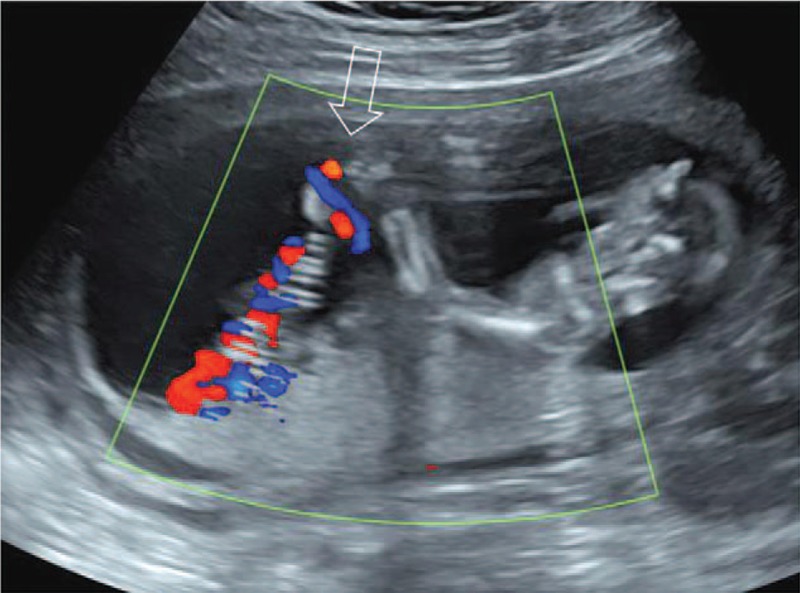
The right hand adhered to the umbilical cord with restricted movement at 13 + 3 gestational weeks of case 1.

**Figure 2:**
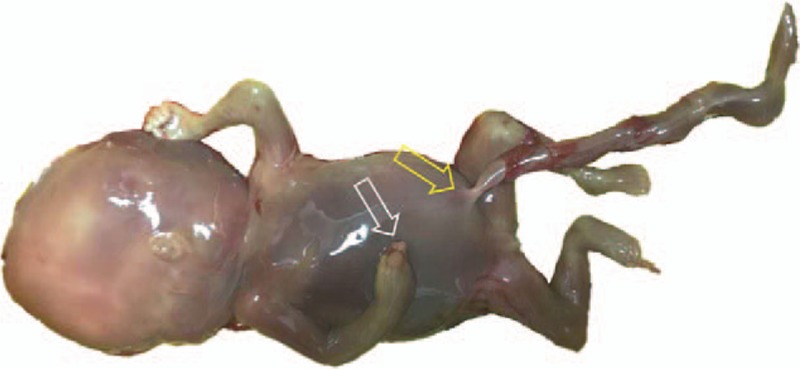
The narrowing of some segments of the umbilical cord (yellow arrow) and deformity of the right fingers (white arrow) of case 1 by pathologically.

A 34-year-old woman, gravida 1 para 0, underwent routine ultrasound examination at 11 to 14 weeks. Her previous history and family history were unremarkable. At the first US examination at 13 + 3 weeks’ gestation, only the fetal hand adhered to the umbilical cord with restricted movement was found, without any floating bands in the amniotic cavity and other structural abnormalities (Fig. 1). During the reexamination at 15 + 4 weeks of pregnancy, multiple band-like echoes were detected in the amniotic cavity, suggesting the occurrence of ABS. Fetal intrauterine death was found at 17 + 3 weeks’ gestation. Pathology found the malformation of the fetal right hand, the surface of the umbilical cord surrounded by amniotic bands, and the diameter of the umbilical cord root became narrow (Fig. 2).

### 2.2 Case 2

A 39-year-old woman, gravida 5 para 0, had a previous history of 4 spontaneous abortions at 7 to 9 gestational weeks, and no family history of ABS. At 12 + 6 weeks of pregnancy, ultrasound examination showed that the right upper limb of the fetus was overflexed, and the right hand was adhered to the umbilical cord with limited movement. Reexamination at 15 + 1 week of pregnancy detected adhesion between both fetal hands and umbilical cord, unclear border of anterior chest wall, and multiple band-like echos in amniotic cavity. Intrauterine death was found at 19 + 4 weeks. After induction of labor, the deformities of both hands, the defect of anterior chest wall, and the adhesion between fetal upper limb and umbilical cord surface by amniotic bands were found.

## 3 Discussion

The ultrasonic manifestations of ABS vary due to different affected fetal parts, from only simple constricted rings to severe morphological and functional abnormalities of craniofacial region, trunk, limbs, and other areas.^[1,2]^ There are 4 types of ultrasound features of 28 ABS cases in the first gestation summarized by Ushakov and Lia,^[3]^ including amniotic net, dividing amnion, amniotic connection and baby in an envelope. But in some cases without the above signs, especially in early pregnancy, it is difficult to diagnose ABS when the fetal abnormalities and the amniotic bands echoes are not obvious. It has been proposed that observing extremity morphology may contribute to the diagnosis of ABS. According to the 10 patients diagnosed with ABS by Barzilay et al,^[4]^ in which 3 cases (30%) had hand amputation, 3 cases (30%) showed hand or foot confined by amniotic bands, and the remaining 4 cases (60%) showed deformed hand or absent fingers. Our 2 fetuses only showed 1 hand adhered to the umbilical cord with fixed position, with no amniotic band-like echoes in the 11 to 14 weeks of gestation, but confirmed of ABS in the follow-up examination. Therefore, observing the movement of the upper limbs and the relationship between hands and umbilical cord can improve the diagnosis rate of ABS.

Fetal prognosis of limb deformity caused by ABS is better than that of fetuses with craniofacial or trunk system involvement.^[1,2]^ However, in some cases of ABS, there was no abnormality detected in prenatal US examination especially in early pregnancy, and it was not until the termination of pregnancy due to intrauterine death that the umbilical cord entangled by the amniotic bands was discovered. We reviewed the Pubmed literature from 1992 to 2018, and totally 11 fetuses whose found umbilical cord entangled by amniotic bands, 8 died in utero and 3 survived.^[2,5–10]^ The poor prognosis of the fetus may be due to compression or constriction of the umbilical cord, which is also the reason of fetal intrauterine death in this group. The fetal hand and umbilical cord were simultaneously adhered by the amniotic bands, which caused the compression of umbilical cord. In the ultrasonic examination of early pregnancy, it is of great significance to dynamically observe whether fetal limb movements and postures change with fetal movements. Those highly suspicious of ABS should be closely followed up.

ABS should be differentiated from other diseases causing fetal limb malformation in early pregnancy. The etiology of fetal limb deformities is complex, such as monogenic diseases, chromosomal abnormalities, intrauterine factors, vascular factors, maternal diseases, drug exposure during pregnancy, among others, but in many cases, the etiology remains unknown.^[4,11]^ In ABS, the residual limb displayed cluttered echoes, irregular shape, and usually unilateral limb abnormality due to the destruction of amniotic bands. Other limb malformations caused by congenital teratogenic factors such as fetal chromosomal abnormalities are usually bilaterally symmetric, which can be distinguished from ABS.^[12,13]^

Our study had some limitations. First, owing to the rarity of ABS, we did not have a large sample. Second, the 2 fetuses did not perform chromosome or genetic examinations. Although no gene mutation and inheritance pattern has been identified that associated with ABS,^[1,2,14]^ the genetic examination are suggested for patients to distinguish other genetic disorders. Finally, due to cultural habits and to reduce the risk of vaginal infection and inflammation, no trans-vaginal US examination was performed during 11 to 14 weeks, and only transabdominal US examination may have limited detection of amniotic band echoes.

In summary, it is significant to observe the shape, posture, and activity of the fetal hand in the early gestation. We propose that the fetal hand adhered to umbilical cord with limited activity is an important sonographic sign of early pregnancy suggesting ABS with poor prognosis, which should be given enough attention during 11 to 14 weeks’ US examination.

## Author contributions

**Conceptualization:** Hua Meng, Yunshu Ouyang, Xining Wu.

**Data curation:** Zihan Niu, Hua Meng, Yixiu Zhang, Xining Wu.

**Formal analysis:** Hua Meng, Yunshu Ouyang.

**Investigation:** Hua Meng, Xining Wu.

**Resources:** Hua Meng, Xiaoyan Zhang, Yixiu Zhang.

**Writing – original draft:** Zihan Niu, Xining Wu.

**Writing – review & editing:** Zihan Niu, Hua Meng.

## References

[1] LópezMuñoz E Becerrasolano LE An update on amniotic bands sequence[J]. Arch Argent Pediatr 2018;116:e409–20. 29756715 10.5546/aap.2018.eng.e409

[2] Larsen LA Umbilical cord strangulation by amniotic bands. J Obstet Gynaecol Can 2018;40:1265. 28870722 10.1016/j.jogc.2017.05.033

[3] Ushakov F Lia C Amniotic band syndrome: first trimester diagnosis and classification. Ultrasound Obstet Gynecol 2017;50:186.

[4] Barzilay E Harel Y Haas J Prenatal diagnosis of amniotic band syndrome—risk factors and ultrasonic signs. J Matern Fetal Neonatal Med 2015;28:281–3. 24735486 10.3109/14767058.2014.915935

[5] Larciprete G Montagnoli C Fusco P Severe fetal distress and umbilical cord strangulation. Case Rep Med 2011;2011:645487. 21785605 10.1155/2011/645487 PMC3139904

[6] Reles A Friedmann W Vogel M Intrauterine fetal death after strangulation of the umbilical cord by amniotic bands. Geburtshilfe Frauenheilkd 1991;51:1006–8. 1794679 10.1055/s-2008-1026254

[7] Elchalal U Ashkenazy M Weissman A Strangulation of the umbilical cord due to combined amniotic band and true knot. Int J Gynaecol Obstet 1992;38:45–7. 1348990 10.1016/0020-7292(92)90729-3

[8] Graf JL Bealer JF Gibbs DL Chorioamniotic membrane separation: a potentially lethal finding. Fetal Diagn Ther 1997;12:81–4. 9218946 10.1159/000264436

[9] Lurie S Feinstein M Mamet Y Umbilical cord strangulation by an amniotic band resulting in a stillbirth. J Obstet Gynaecol Res 2008;34:255–7. 18412792 10.1111/j.1447-0756.2008.00765.x

[10] Chatzigeorgiou K Theodoridis T Efstratiou I Strangulation of the umbilical cord by an amnion band - a rare cause of intrauterine demise: a case report. Cases J 2009;2:9108. 20062685 10.1186/1757-1626-2-9108 PMC2803905

[11] Andrikopoulou M Vahanian SA Chavez MR Improving the ultrasound detection of isolated fetal limb abnormalities. J Matern Fetal Neonatal Med 2017;30:46–9. 26932755 10.3109/14767058.2016.1160048

[12] Tadmor OP Kreisberg GA Achiron R Limb amputation in amniotic band syndrome: serial ultrasonographic and Doppler observations. Ultrasound Obstet Gynecol 1997;10:312–5. 9444043 10.1046/j.1469-0705.1997.10050312.x

[13] Higuchi T Tanaka M Kuroda K Abnormal first-trimester fetal nuchal translucency and amniotic band syndrome. J Med Ultrason 2012;39:177–80. 10.1007/s10396-012-0344-0 27278979

[14] Moran SL Jensen M Bravo C Amniotic band syndrome of the upper extremity:diagnosis and management. J Am Acad Orthop Surg 2007;15:397–407. 17602029 10.5435/00124635-200707000-00005

